# Systematic Evaluation of Biologically Inspired Motion Detection Models: From LGMD and EMD to Hybrid Spiking Neural Networks

**DOI:** 10.3390/biomimetics11060374

**Published:** 2026-05-28

**Authors:** Vanessa Ndiangang, Pengcheng Liu

**Affiliations:** Department of Computer Science, University of York, York YO10 5DD, UK

**Keywords:** motion perception, collision detection, dynamic scenarios, bio-inspired robotics, insects, spiking neural networks

## Abstract

Collision detection in dynamic environments demands perception systems that are both computationally efficient and robust to diverse motion patterns. Biological vision systems, particularly those of insects, offer efficient neural architectures capable of rapid motion interpretation under strict resource constraints. This work presents a systematic comparative evaluation of three biologically inspired models: the Lobula Giant Movement Detector (LGMD), the Elementary Motion Detector (EMD), and a hybrid Spiking Neural Network (SNN) incorporating LGMD and EMD-derived motion processing pathways, evaluated on programmatically generated synthetic stimuli with frame-level ground truth. The hybrid SNN achieved an accuracy of 73–87% across stimulus types, consistently exceeding the 75.0% held-out test set baseline, with a precision of 1.0 throughout and a substantially lower runtime than the LGMD implementation. LGMD demonstrated rate-based sensitivity consistent with biological spike-frequency adaptation, while the EMD correctly produced near-zero responses to looming stimuli, confirming its role as a directional rather than collision detector. These results demonstrate that hybridising biologically inspired motion detectors within a trainable spiking framework produces a promising and reproducible approach to collision prediction, while identifying the sim-to-real generalisation gap as a key challenge for future deployment.

## 1. Introduction

Collision detection is a critical functionality of robotic systems. It enables the necessary counteraction against obstacles, edges or other elements in the system [1]. A robust collision detection mechanism is a crucial underpinning in dynamic systems, in which objects move at varying speeds, light is emitted with varying intensities and directions and noise or interference can cause interruptions or misreadings by sensors [2]. The proliferation of this function increases in such environments [2,3], more so than those that are static, escalating with an indefinite number of elements, including an agent’s motion, location and relation to its surroundings or other robotic agents.

Dynamic environments impose several constraints and limitations. The fundamental unpredictability of dynamic obstacles [4] remains a core challenge. The need for real-time processing [3] often pushes the boundaries of available hardware and computational resources, particularly for smaller robots [4]. Achieving robust navigation requires effective sensor integration and algorithm implementation [3], which can be particularly challenging in dynamic settings. Furthermore, adapting existing navigation approaches designed for static environments to handle dynamic elements is a non-trivial task [3]. In multi-robot systems, the added layer of effective communication and coordination in a dynamic environment [3] introduces further complexity. Moreover, real-world validation through experiments in diverse and complex environments is often lacking [3]. Static testing environments do not effectively account for high-velocity objects. Bio-inspired models, which are driven by complex environments, appear to achieve robustness and reliability [2].

Biological systems and organisms can be considered as models that are particularly well suited to tackle collision detection in dynamic environments [5]. Systems derived from the visual perception or neural processing capabilities of organisms such as insects are particularly advantageous in this regard [5,6]. The structures in biological organisms informing functionally equivalent computational models often are found in cluttered or dense dynamic environments, appearing to enable the organisms in collision perception [3]. Organisms such as insects are thought to have organically developed structures that are computationally simple, efficient and robust. Typically, non-biological models exhibit reduced performance under conditions of high scene complexity and constrained computational resources [3]. This, in turn, positions biological models well for assimilation with other structures with complementary functionalities, enabling further efficacy [7].

Research into the visual neural networks of organisms such as insects has resulted in the development of visual collision detection models [3,5]. These models are particularly useful for their computational efficiency [8], robustness to dynamic scenarios [2], timeliness [8] and high collision success rates [5]. Lobula giant motion detectors (LGMDs), identified in locusts, display specific sensitivity to imminent motion cues and modulations of light intensity [2]. Elementary motion detectors (EMDs), also derived from insect vision systems, are proficient in directional motion encoding and utilise optic flow, a low-level motion signal, to perceive object paths [3]. Spiking neural networks (SNNs) provide a flexible, brain-inspired temporal encoding framework [9].

Evaluating and potentially combining well-developed and researched aspects of biological structures, such as LGMDs, EMDs, and SNNs, for collision detection, particularly in realistic, challenging, dynamic scenarios, is crucial for addressing several core research problems in the development of robust and efficient artificial vision systems. A traditional collision detection model may have higher computational overhead than that observed in biological models [1], such as LGMDs and EMDs, as these have been shown to function primarily in low-resource systems [4]. The optimised energy and hardware utilisation further enable them to be used in cost and resource-efficient neuromorphic sensors [5]. Recent work has further demonstrated the potential of bio-inspired visual systems for collision avoidance and multi-object detection in dynamic environments [10,11].

The primary objective of this paper was to explore the effectiveness of biologically inspired models for visual collision detection in dynamic environments. Specifically, the paper focused on three models: the Lobula Giant Movement Detector (LGMD), the Elementary Motion Detector (EMD), and a custom-built hybrid Spiking Neural Network (SNN) that integrates core principles from both. The study aimed to evaluate each model under identical testing conditions using programmatically generated synthetic stimuli with frame-level ground truth. Additionally, it aimed to compare their performance in terms of accuracy, precision, recall, F1-score, and spike-based activity metrics across five synthetic stimulus types, and to characterise the biological sensitivity profile of each model under controlled evaluation conditions. The source code and trained model weights are openly available at [12].

This article is organised into six main sections. The introduction outlines the context and motivation for the study, explaining the relevance of biologically inspired vision models in robotic collision detection. The literature review surveys the foundational principles and previous research on LGMD, EMD, and SNN systems, highlighting their respective capabilities and limitations. The Section 3 details the dataset design, model implementations, and evaluation approach, ensuring consistency and fairness across comparisons. The Section 4 presents the outcomes of the experiments, comparing model performance across multiple metrics and scenarios. The discussion reflects on the findings, explores their implications, and considers the practical deployment of each model. Finally, the conclusion summarises the key contributions and proposes directions for future work. Supplementary Materials, including implementation code at [12] are provided to assist in result interpretation and reproducibility.

## 2. Related Works

### 2.1. Foundational Principles

The structures of and within biological organisms have inspired a range of computational principles that are increasingly applied in collision detection. Optic flow refers to the motion perceived by a vision system. In translational motion, close-by objects induce large optic flow amplitudes and far-away objects appear to move more slowly [13]. EMDs are typically computational structures used to determine optic flow. Motion pattern recognition is another principle typically used in robotic systems that can be computationally modelled from biological structures. EMDs are directionally selective neurons (DSNs) as they are capable of detecting translational movement [7]. To detect the motion of objects in and out of depth, LGMDs employ looming detection [5]. This emphasised selectivity to motion features is characteristic of biological visual systems in particular [8].

The biological principle of parallel processing, for instance, of luminance increments (ON) and decrements (OFF) in separate, parallel pathways, is also built into EMDs and LGMDs. These parallel pathways map onset and offset responses to changes in light intensities perceived [8] and process translational or directional motion captured by photoreceptors in parallel. They are useful for their computational efficiency, particularly in mass and power, for their robustness in complex environments, for their biological plausibility and for their prominence in preliminary visual signalling. Biological ephemeral vision pathways are applied to dynamic vision sensors (DVS), which are then used in event cameras to facilitate an asynchronous reaction to light intensity variation [8]. DVS are able to benefit from the inherently low latency, high signal range, and high temporal resolution to process high-speed movements in dynamic environments [8].

Insects such as locusts and flies are considered to be experts in motion perception as they possess complex visual systems that can effectively segment visual stimuli and facilitate a unified response [14,15]. Photoreceptors [2] that detect visual stimuli initiate transduction that converts the stimuli to electrical signals to be interpreted by their nervous systems, which comprises circuits and neural networks dedicated to the comprehension of motion. This capability allows insects to discern, prioritise, and respond appropriately to different potential collision scenarios based on their type, the risk they pose and the relevance to the task at hand. Broadly, research has been able to establish a relationship between neuroethology and computing, specifically between the perception evaluation circuits found in organisms and the robotic control systems using biological principles to enhance robot navigation, collision avoidance and reactive decision-making.

### 2.2. The Task of Collision Detection

Collision detection is a procedure that processes information about a robot and its environment to determine if a collision has occurred or is likely to occur. Such algorithms generally aim to detect approaching objects on a collision course and take timely and accurate action to prevent a collision [13]. In real time, the algorithm can be comprised of two phases: broad-phase and narrow-phase [16]. Broad-phase quickly identifies likely collisions, and narrow-phase accurately confirms and details them [16]. To avoid collisions, visual motion cues, biological principles, including optic flow, motion pattern recognition, parallel input processing and ephemeral vision pathways, among others, are applied to collision avoidance. Its success can be evaluated according to the following: latency [17], energy consumption [17], computational efficiency [17], sensitivity [8], accuracy [1], robustness [3] and adaptability [18]. Specifically, low latency and energy consumption, as well as high energy consumption, computational efficiency, sensitivity, accuracy and robustness will be characteristic of successful mechanisms.

### 2.3. Bio-Inspired Models

#### 2.3.1. LGMD (Lobula Giant Movement Detectors)

LGMDs are interneural networks that enable locusts to anticipate the motion of incoming objects [3,13]. They can be located in the optical lobe of locusts [7,8] and function as wide-field motion detectors [7,8]. Excitatory and inhibitory signals in the LGMD circuitry respond to the acceleration of an object towards locusts, which are perceived as fast objects, growing as they approach them. Two forms of the interneurons, LGMD1 and LGMD2, are distinguished by their functional roles, connectivity within the locust visual systems and modes of response. LGMD1 implement signalling from ON and OFF pathways, that is, to both light and dark objects that are approaching the organism. On the other hand, LGMD2 appear take on the nature of OFF pathways, responding to light-to-dark transitions, notably in bright conditions [5].

LGMD-based models have been applied in a range of practical and experimental contexts [3]. In real-time scenarios, these models may be deployed for collision detection in mobile micro-robots using vision systems. In simulated environments, similar approaches apply to ground vehicle vision tasks. Notably, combined LGMD1 and LGMD2 models are employed to simulate traffic scenarios, supporting more accurate collision prediction. The Synthetic Neural Vision System, incorporating models of LGMDs and Fly Direction Selective Neurons (DSNs) [19], is applied to motion pattern recognition in micro-robots, specifically for collision avoidance and tracking tasks. Its performance is evaluated through open-loop tests, measuring the spike frequency of the artificial neurons in response to motion, and arena tests, assessing the success rate of collision avoidance in dynamic robot interactions.

There are some limitations that have been observed in LGMD models. Parameter setup requires high fidelity models as the data used to initialise the parameters is obtained experimentally in biological settings [8]. While most models are computationally minimalistic, they increase in complexity when implemented in micro-robot settings [2,3]. Current testing is also lacking validity due to the few tests in real-world environments. LGMD-based collision avoidance may lead to less direct robot paths [13], struggle with edge detection in the arena [7], and fail to effectively respond to overhead obstacles or ground shadows [3]. Such limitations limit the scope within which LGMDs can function and facilitate collision detection.

#### 2.3.2. EMD (Elementary Motion Detectors)

EMD [8] is a theoretical computational model of the optic flow mechanism in insects that computes local directional motion perceived by photoreceptors. They are most well-suited to perceive translational motion, in which close-by objects induce large optic flow amplitudes and faraway objects appear to move more slowly [13]. They are also useful for perceiving objects of different sizes because they can be set to detect different spatial frequencies [4]. This mechanism works by comparing changes in brightness over space and time using nearby photoreceptors, often incorporating ON/OFF channels, enhancing stimulus segmentation, particularly in resistance to background noise [8,20]. Excitatory signals are sent to neurons by translation in one direction of motion and inhibitory signals by the opposite direction [5]. This spatio-temporal signal correlation [21] usually occurs between a minimum of two photoreceptors and relies on delays between these signals to model optic flow [8].

In organisms and robots alike, they are involved in landing, stabilisation in flight, navigation through corridors and across terrain, and notably, in collision detection [5]. Models inspired by EMDs in combination with Small-Target Motion Detector (STMD) found in insect visual systems [3] are being applied to the challenge of tracking small, moving targets against complex, cluttered backgrounds, particularly in mobile ground vehicles. The utility of such models lies in their ability to reliably track small, moving objects even when the background is visually noisy and distracting.

Interestingly, they are not data-driven and are instead model-driven [8]. Their rapid response rates [17], parsimony, and computational efficiency make them suitable for micro-robots or in low hardware and computational resource environments [2]. However, similarly to LGMDs, EMDs lack effective feedback transmission, which decreases accuracy and negates learning capabilities [8].

#### 2.3.3. Spiking Neural Networks (SNNs)

SNNs are described as the third generation of artificial neural networks [9]. In comparison to other neural network frameworks, SNNS are simpler, more accurate and more adaptable, and more suitable for real-time applications with restrictions on computing resources [17]. They model individual neurons using computational units like Leaky-Integrate Fire (LIF) or Adaptive Exponential neurons, inspired by those in the mammalian visual cortex [6]. These structures feature a membrane potential that builds up from incoming signals and triggers a spike once it crosses a certain threshold [9]. SNNs aim to mimic how the brain uses spike timing (including when spikes occur and at what rate, potentially across many neurons) to encode information, communicating through sequences of these spikes called spike trains [9,22]. SNNs emulate the sparse and event-driven communication found in the brain, where neurons only activate when communication is established, primarily based on the transmission of these discrete spikes [9,22]. Finally, similar to the brain’s hierarchical processing through numerous layers of interconnected neurons [22], Deep SNNs are designed with multiple layers to enable complex feature extraction and processing [20].

Their low energy consumption due to organic spike timing inspired by that of the brain [17] contrasts artificial neural networks (ANNs), which are underpinned by continuous-weighted activations [22]. Additionally, in comparison to ANNs, SNNs process information in close timing with stimulus perception, offering low latency, although at times decreasing in accuracy [20]. Furthermore, SNNs are uniquely adapted to, in complex, dynamic environments, parse information encoded in spikes [9], particularly in online computations [20]. What remains is to standardise training algorithms for SNNs [17], apply them to higher-level tasks [23], evaluate and test them against other biological computational structures and models and extend them to deep learning [17].

### 2.4. Comparison, Hybridisation and Future Directions

The biological grounding and computational implementation of LGMDs, EMDs and SNNs differ. While LGMDs are high-fidelity physiological models of a biological structure, EMDs are more so a computational structure inspired by biological structures. In real-world applications, LGMD and EMD models differ notably in the types of motion they detect, i.e., looming for LGMDs and lateral in the case of EMDs [8]. In real-world robotics applications, this often enables the models to be deployed complementarily [7], such that impending collision and translational motion signals can be perceived as a unit of computation. Tangentially, the neuroethological spike-based nature that underpins the SNNs’ information communication procedure [17] may be able to extend the capabilities of itself and other bio-inspired computational models when providing the mechanical basis for them.

The computational models share biological principles, including parallel processing of ON/OFF pathways, directional selectivity and feed-forward processing. However, as shown in Table 1, the models diverge in their movement processing selectivity, neural computation mechanisms, learning strategies and network complexity [8,17]. SNNs are more complex in these regards than LGMDs and EMDs. Relatively simplistic bio-inspired models like LGMD and EMD, while inspired by complex biological neurons, are often computationally simplified to make them practical for real-world applications, including embedded neuromorphic systems [5]. Their strength lies in their resource efficiency for dedicated tasks [8]. However, challenges regarding accuracy in complex dynamic scenes are a pervasive issue.

SNNs appear to provide a promising framework [17] within which computational models such as LGMDs and EMDs may be able to be combined and developed to communicate visual motion signals, in potentially more complex, hardware and resource constrained environments [2,8] while maintaining reasonable proximity to biology and computing [20]. Numerous studies support the integration of LGMDs and EMDs into SNNs to enhance robustness in real-world, dynamic environments. Research has tested the implementation of LGMDs and EMDs on real-time, embedded systems for movement and perception tasks [5]. Such hybridisation has already been shown to improve accuracy in cluttered scenes and enhance responsiveness to varied motion cues [24].

A hybrid LGMD-SNN model could embed the core architecture of the LGMD, photoreceptors, lateral inhibition, and spike generation within a spiking neural framework. Each layer in the LGMD could be implemented using spiking neurons such as LIF units, enabling the system to maintain biological fidelity while also supporting integration into larger SNN pipelines. One design might treat the LGMD structure as a feature extractor within a broader SNN, feeding collision-related spiking events into downstream decision-making or control layers. Alternatively, LGMD and EMD streams could function as parallel pathways within a spiking architecture, with integration or competition occurring in higher layers. This architecture allows for improved selectivity (e.g., LGMD1 for light/dark, LGMD2 for dark-only), modularity, and online learning in environments with fluctuating complexity [3]. Another design could combine EMDs with deep SNNs, introducing the potential for hierarchical motion feature extraction [22], where EMD outputs can be processed by deeper spiking layers to support complex visual recognition tasks such as trajectory estimation or obstacle tracking. This could significantly expand the utility of EMDs beyond simple motion detection, especially when paired with event-based sensors or neuromorphic cameras [6]. Similarly, integrating feedback mechanisms into the LGMD-based SNNs (as in F-LGMD variants) can enhance adaptability and robustness. Feedback can help modulate gain control, suppress irrelevant motion, and support context-sensitive collision detection. These hybrid systems more closely mirror biological circuits and reduce the need for hand-tuned thresholds or hard-coded logic, making them suitable for deployment in real-world, reactive control tasks [17].

The implementation and evaluation of these biologically inspired models contribute meaningfully to the advancement of adaptive and efficient robotic systems across a range of applications. LGMD-based architectures, for example, are particularly effective for rapid frontal obstacle detection, making them highly suitable for use in UAVs and micro-robots where low-latency collision avoidance is essential [4]. In parallel, EMD-inspired models provide robust perception of translational motion and optic flow, which are foundational for navigation tasks such as obstacle avoidance, visual odometry, and landing control in both aerial and ground-based robotic platforms [8]. The integration of these vision models within energy-efficient SNNs, especially when deployed on neuromorphic hardware [9], enable scalable and real-time control under power constraints, offering a compelling solution for autonomous vehicles and swarm robotics where efficiency is paramount [25]. Moreover, incorporating EMD or LGMD modules into multi-agent robotic systems supports decentralised coordination by leveraging local visual cues for inter-agent collision avoidance, thus enhancing group-level intelligence without the need for centralised oversight [3].

LGMDs and EMDs demonstrate computational efficiency and robustness, particularly in low-resource or cluttered settings, but are inherently limited by fixed architectures and threshold dependencies. SNNs can extend these capabilities by supporting sparse event-based communication and hierarchical processing of spatial-temporal information. By comparing LGMD, EMD, and a hybrid SNN that integrates their mechanisms, it becomes possible to evaluate trade-offs in accuracy, responsiveness, and deployability. Such a comparative study is motivated by the shared biological inspiration, differing sensitivities to motion types, and the increasing demand for scalable, neuromorphic solutions in real-world autonomous navigation. This study aims to critically investigate the extent to which these biologically inspired models, LGMD, EMD, and a hybrid SNN, can contribute to robust and efficient collision detection in visually dynamic environments. By systematically evaluating their performance on shared datasets and comparing their outputs across key metrics such as responsiveness, accuracy, and resource efficiency, the research explores not only the relative strengths and limitations of each approach but also the potential advantages of integrating biologically grounded architectures within trainable, spike-based neural frameworks. In doing so, the study seeks to establish how well such models scale under realistic visual conditions and what role hybridisation plays in advancing biologically inspired vision in dynamic scenarios.

## 3. Materials and Methods

### 3.1. Overview of the Approach

This paper evaluates three biologically inspired motion detection models: the LGMD2, the Elementary Motion Detector (EMD), and a hybrid Spiking Neural Network (SNN), under identical controlled conditions using programmatically generated synthetic stimuli. Performance is assessed in terms of accuracy, precision, recall, F1-score, and spike-based activity metrics, with particular attention to the biological sensitivity profile of each model. The hybrid SNN integrates LGMD-inspired parallel ON/OFF convolutional pathways for looming detection with EMD-inspired spatiotemporal integration for directional motion sensitivity, implemented within a trainable spiking framework using Leaky Integrate-and-Fire (LIF) neurons [9]. This architecture was selected to combine the fixed biological selectivity of LGMD and EMD with the adaptability of learned spike-based representations [17], enabling direct comparison between hard-coded biological models and a trainable bio-inspired hybrid under shared evaluation conditions [7].

### 3.2. Model Implementation

#### 3.2.1. LGMD Model (*C# and .NET*)

LGMD neurons are typically modelled as LGMD1, LGMD2, or hybrid variants, often incorporating ON and OFF pathways to support parallel processing and improve robustness in complex dynamic environments. Details are shown in Table 2. For this study, the LGMD2 model was selected due to its biological specialisation and its relatively modular structure, which made it more suitable for integration into a frame-by-frame video processing pipeline. This model was implemented using an open-source *C#* version [26] based on the original work described in Fu et al. [3]. The LGMD2 model was implemented in *C#* using the *.NET 8.0 framework*, with video processing handled via the *OpenCvSharp* library. Additional *.NET* libraries such as *System.Drawing.Common* supported image handling, and the model was compiled and executed using the dotnet CLI. The model was initialised with biological parameters derived from the original implementation [3], including a spike-frequency adaptation time constant of $\tau_{sfa}=800$ ms, a spiking threshold of $T_{sp}=0.78$, and a collision detection criterion of $N_{ts}=8$ cumulative spikes. These parameters were not modified from the original implementation, preserving biological fidelity.

The LGMD2 model produces several outputs per video frame to reflect the internal state and decision-making of the simulated visual neuron, as shown in Table 3. These include the membrane potential, which represents the neuron’s activation in response to motion stimuli, and the spike output, indicating whether the neuron fired and how strongly. A binary collision detection signal flags whether a looming object has been identified, serving as the primary output for evaluating the model’s performance on collision tasks. Additional outputs include the cumulative spike count, which helps assess energy efficiency, and the motion energy, a value derived from the photoreceptor layer that indicates input intensity. The spike-frequency adaptation (SFA) parameter reflects how the model dynamically adjusts spiking behaviour based on recent activity, mimicking biological habituation. These outputs provided a compact yet expressive snapshot of the model’s temporal response, suitable for both visualisation and quantitative analysis [9,17].

Early in testing, the demonstration videos packaged with the LGMD source code [26] failed to elicit meaningful collision responses, as they were designed to visualise internal model layers rather than simulate functional collision scenarios. This observation motivated the development of a purpose-built synthetic stimulus set with programmatic ground truth, described in Section 3.3. The LGMD model was evaluated on all five stimulus types using the frame-by-frame processing pipeline, with the binary Collision output used as the primary frame-level prediction for evaluation purposes. A key finding during evaluation was that the LGMD fired at frames 9–11 of looming stimuli, before the programmatic collision threshold of radius greater than 16 pixels was reached. This reflects the model’s biological sensitivity to expansion rate rather than expansion magnitude, consistent with spike-frequency adaptation observed in biological LGMD circuits [3].

#### 3.2.2. EMD Model (Python)

The Elementary Motion Detector (EMD) model is grounded in the Reichardt correlation mechanism [27], a well-established framework in computational neuroscience used to explain directional motion perception in insects. The original MATLAB-based implementation [28] was not used in this study due to its dependency on Simulink model files unavailable in the current environment. Instead, a simplified but functionally equivalent Reichardt correlator was implemented directly in Python 3.14.3, capturing the core biological mechanism without the Simulink overhead.

The correlator operates on consecutive grayscale frame pairs. For each pair, the central horizontal row of pixels (row 32 of a 64 × 64 frame) is extracted, corresponding to the horizontal midline where disc motion is most informative. Directional motion energy is computed by comparing temporally delayed signals between adjacent photoreceptors. Specifically, the luminance of each pixel in the previous frame is multiplied by the luminance of its right neighbour in the current frame, summed across all adjacent pairs to produce a rightward motion signal. The same operation is performed in reverse to produce a leftward motion signal. The difference between these two signals yields a signed scalar motion energy value per frame: positive for rightward motion, negative for leftward, and near zero for symmetric expansion or no directional motion.

**Figure 1:**
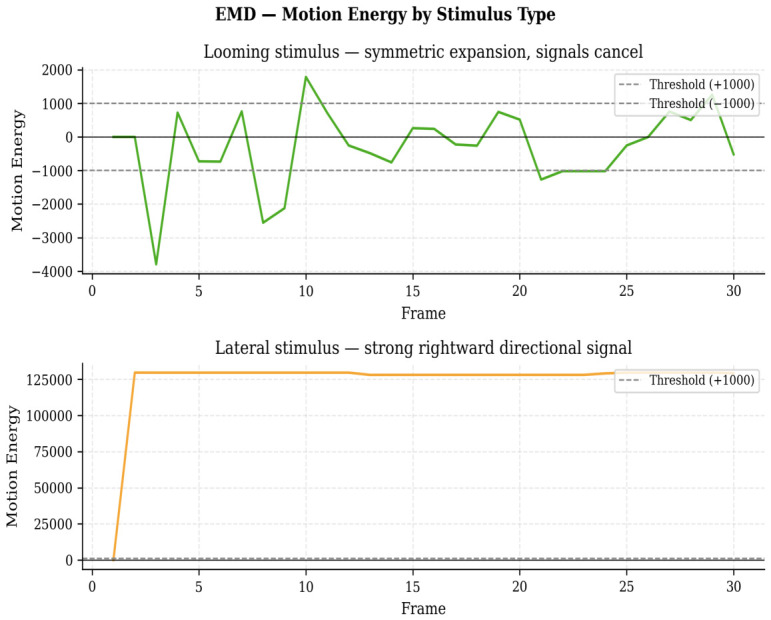
EMD motion energy for looming and lateral stimuli. The looming stimulus produces near-zero energy as symmetric radial expansion cancels opposing directional signals. The lateral stimulus produces a strong positive signal confirming rightward motion detection. This validates the Reichardt correlator implementation prior to evaluation.

This implementation was validated against a priori biological predictions before evaluation. As shown in Figure 1, looming stimuli, which produce symmetric radial expansion, yielded a mean motion energy of 0.00, confirming cancellation of opposing directional signals. Lateral stimuli yielded a mean motion energy of 112,112, confirming a strong rightward directional response. These results are consistent with the known biological role of EMDs as directional motion detectors rather than collision detectors [8].

For binary prediction, a motion energy threshold of 1000 was applied. This threshold was selected to reflect the EMD’s biological role as a detector of any meaningful directional motion signal, rather than requiring collision-level magnitudes that the symmetric cancellation mechanism cannot produce for looming stimuli. The threshold is, therefore, conservative by design, consistent with the EMD’s function as a motion-sensitivity indicator rather than a collision classifier.

#### 3.2.3. Hybrid SNN Model Implementation (Python/SpikingJelly)

The hybrid Spiking Neural Network (SNN) was developed using the SpikingJelly framework (version 0.0.0.0.14) [29] and implemented in Python using PyTorch 2.12.0. The architecture integrates two biologically motivated processing streams within a trainable spiking framework. Parallel ON and OFF convolutional pathways, each consisting of an 8-filter Conv2d layer followed by a Leaky Integrate-and-Fire (LIF) neuron layer, are inspired by the LGMD2 ON/OFF pathway structure. The ON pathway processes the input directly, while the OFF pathway processes the luminance-inverted input, replicating the biological separation of light increment and decrement signals. The outputs of both pathways are concatenated and passed through a 16-filter merge convolutional layer with a further LIF layer, implementing spatiotemporal integration inspired by EMD directional correlation. An adaptive average pooling layer reduces spatial dimensions to a single value per channel, which is passed to a fully connected output layer, producing a scalar logit for binary collision prediction.

All LIF neurons share the parameters τ=2.0 (membrane time constant) and $v_{threshold}=0.2$ (firing threshold). Input frames are normalised to the range [0, 1] by dividing by 255, then scaled by a factor of 5.0 to drive membrane potentials above the firing threshold. The network processes sequences of 10 consecutive frames, formatted as tensors of shape (10, 1, 64, 64), where each timestep *t* receives a distinct frame rather than a repeated input. The network state is reset between sequences using functional.reset_net().

**Figure 2:**
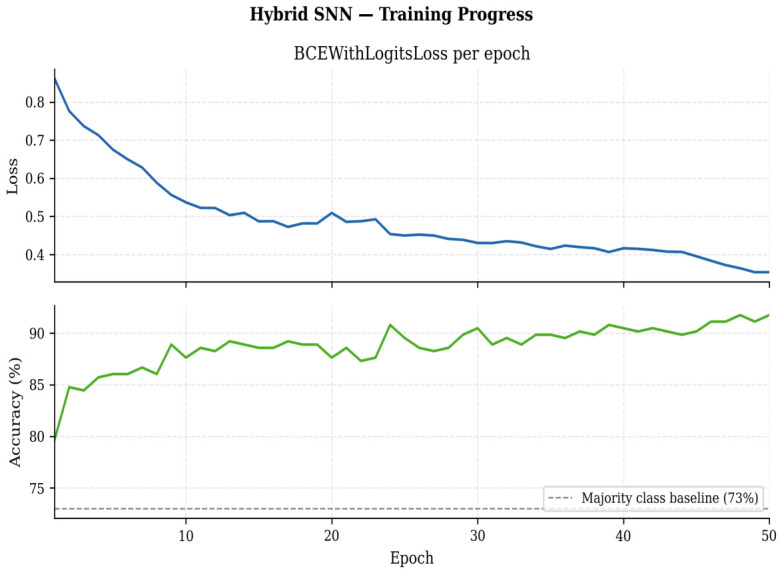
Hybrid SNN training progress over 50 epochs. Loss decreases consistently from epoch 1 to epoch 50. Accuracy exceeds the 73% majority class baseline from epoch 1 and stabilises above 90% by epoch 30.

The hybrid SNN hyperparameters and training configuration is detailed in Table 4. The model was trained on the synthetic stimulus dataset described in Section 3.3 using Binary Cross Entropy with Logits loss (BCEWithLogitsLoss) and the Adam optimiser with a learning rate of $1\times 10^{-3}$. To address class imbalance (230 negative sequences versus 85 positive sequences), a positive class weight of 2.7 was applied, computed as the ratio of negative to positive examples (230/85 = 2.7). As shown in Figure 2, training was conducted for 50 epochs using online learning, with weights updated after each sequence. Training loss decreased consistently from 1.02 at epoch 1 to 0.45 at epoch 50, with final training accuracy of 88.9%, representing a 15.9 percentage point improvement over the 73.0% majority class baseline. Peak accuracy of 88.9% was observed at epochs 37, 41, 44, 45, 47 and 49. Trained weights were saved and loaded for all subsequent evaluation runs, ensuring that inference used learned rather than random representations.

To enable unbiased evaluation, the dataset was divided into a training split comprising variants 1–3 of each stimulus type (15 videos, 315 sequences) and a held-out test split comprising variants 4–5 (10 videos, 210 sequences). The SNN was trained exclusively on the training split and evaluated on the test split. The LGMD and EMD models, having no trainable parameters, were evaluated on the test split only.

### 3.3. Input Data and Stimuli

This study used programmatically generated synthetic video stimuli to evaluate biologically inspired visual motion models in the context of looming object detection. Synthetic stimuli were selected over naturalistic video datasets to enable precise, frame-level ground truth labelling and to eliminate confounds introduced by background complexity, variable lighting, and subjective annotation. All stimuli were generated using OpenCV in Python and saved as grayscale MP4 files at a resolution of 64 × 64 pixels and a frame rate of 20 fps.

#### 3.3.1. Stimulus Design

Each stimulus consists of a black disc on a white background whose spatial properties change across 30 frames according to the motion type being simulated. Five stimulus types were generated, covering the core motion conditions relevant to each model’s biological design assumptions. Multiple variants of each type were generated with varying parameters to increase dataset diversity. Table 5 summarises the stimulus types, their parameters, and their ground truth label rules.

**Figure 3:**
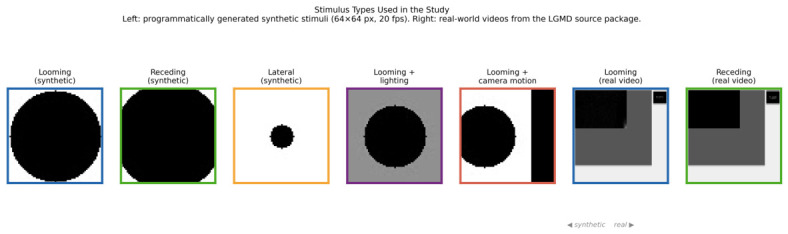
Representative frames from each stimulus type used in the study. Synthetic stimuli (**left five**) comprise a black disc on a white background at 64 × 64 pixels and 20 fps, generated programmatically with known frame-level ground truth. Real videos (**right two**) are taken from the LGMD2 open-source package [3] and contain naturalistic scene complexity absent from the synthetic set. The domain gap between these two distributions is a key factor in the sim-to-real generalisation challenge discussed in Section 5.6.

In addition to the synthetic stimuli, ten real-world videos from the LGMD2 open-source package [3] were included to assess generalisation beyond the synthetic domain. Five videos depict looming objects and five depict receding objects as shown in Figure 3. Unlike the synthetic stimuli, real videos have no per-frame ground truth: each video carries a single video-level label (1 for looming, 0 for receding) applied uniformly across all frames. Real videos were not used for SNN training; they served exclusively as a test set to probe the sim-to-real generalisation gap. Frame dimensions and content vary across real videos (274–354 frames, naturalistic scenes) and differ substantially from the 64 × 64 binary pixel statistics of the synthetic set.

#### 3.3.2. Ground Truth Labelling

Collision imminence was defined as the point at which the expanding disc radius exceeds 16 pixels, representing one quarter of the 64 pixel frame width. This threshold is grounded in LGMD neurophysiology: LGMD does not detect physical contact but responds to the rate and magnitude of visual expansion, initiating escape responses when an approaching object’s angular subtension exceeds a biologically meaningful proportion of the visual field [3]. The threshold was applied programmatically at stimulus generation time, producing frame-level binary labels (1 = collision imminent, 0 = no collision) without manual annotation. Labels were saved as CSV files alongside each stimulus video, with columns Frame, TrueLabel, and StimulusType.

For looming stimuli of 30 frames with a radius growing from 2 to 30, the collision label switches from 0 to 1 at frame 15, yielding approximately 15 positive and 15 negative frames per clip. Receding and lateral stimuli are labelled 0 throughout, as neither stimulus type represents an imminent collision regardless of disc size or position.

#### 3.3.3. Dataset Composition

The full dataset comprised 25 stimulus videos across five types, with five variants per type generated using varying parameters. Across all videos, the dataset contained 750 frames total, with 150 positive (collision) frames and 600 negative frames. After sliding window segmentation into sequences of 10 frames, the full dataset comprised 525 sequences.

To enable unbiased evaluation, the dataset was divided into a training split and a held-out test split based on variant number. Variants 1–3 of each stimulus type formed the training split (15 videos, 315 sequences, 85 positive and 230 negative), yielding a training majority class baseline of 73.0%. Variants 4–5 formed the held-out test split (10 videos, 210 sequences, 50 positive and 160 negative), yielding a test majority class baseline of 75.0%. The SNN was trained exclusively on the training split. The LGMD and EMD models, having no trainable parameters, were applied directly to the test split without any training or tuning.

#### 3.3.4. Preprocessing Pipeline

All video inputs were converted to grayscale to align with luminance-based processing in biological vision systems. Each model received a preprocessed version of the same stimulus videos, adapted to its architectural requirements. The LGMD model processed raw grayscale frames at full 64x64 resolution, capturing pixel-level intensity changes across time. The EMD model extracted the central horizontal row of pixels from each frame to construct a 1D temporal signal for correlation-based motion detection. The hybrid SNN received frame sequences formatted as spatiotemporal tensors of shape (10, 1, 64, 64), normalised by dividing pixel values by 255 and scaling by 5.0 to drive LIF neuron membrane potentials above the firing threshold. All inputs were temporally aligned across models to ensure direct performance comparison across identical visual stimuli.

### 3.4. Evaluation Metrics

Each model outputs a CSV file containing frame-by-frame values corresponding to its internal activity and predicted responses. Table 6 presents multi-model output format, showing per-frame outputs, types, and threshold applications. While the exact variables differ slightly depending on the model, a standard format is followed across all outputs to ensure compatibility during analysis. Typical fields include Frame, SpikeRate, MembranePotential, MotionEnergy, and Prediction.

To evaluate the models consistently, a unified Python evaluation script (evaluate _all_models.py) runs all three models on each stimulus video sequentially, loads the corresponding programmatic ground truth labels from the generated CSV files, and computes frame-level performance metrics. Outputs are stored in model-specific directories and matched frame-by-frame against ground truth labels. Key metrics computed include accuracy, precision, recall, F1-score, true positives, false positives, and false negatives. A majority class baseline is computed for each stimulus type and reported alongside model metrics to contextualise performance. The script also produces a per-frame results file enabling detailed analysis of model behaviour across individual frames and stimulus types.

### 3.5. Summary of Methodology

This methodology establishes a unified, reproducible framework for evaluating three biologically inspired motion detection models, LGMD2, EMD, and hybrid SNN, using programmatically generated synthetic stimuli with frame-level ground truth. Each model is executed independently on identically preprocessed video inputs, with outputs unified into a consistent CSV format for direct comparison. Ground truth labels are generated programmatically at stimulus creation time, eliminating subjective annotation and enabling genuine frame-level evaluation. A majority class baseline is reported explicitly to contextualise all performance claims. Key limitations of this methodology are acknowledged: evaluation is conducted on synthetic stimuli only, runtime comparisons across models are indicative rather than controlled due to differences in implementation language, and energy efficiency is inferred from spike-based metrics rather than measured directly on hardware. These limitations are discussed further in Section 5.6.

## 4. Results and Analysis

### 4.1. Quantitative Performance Evaluation

**Figure 4:**
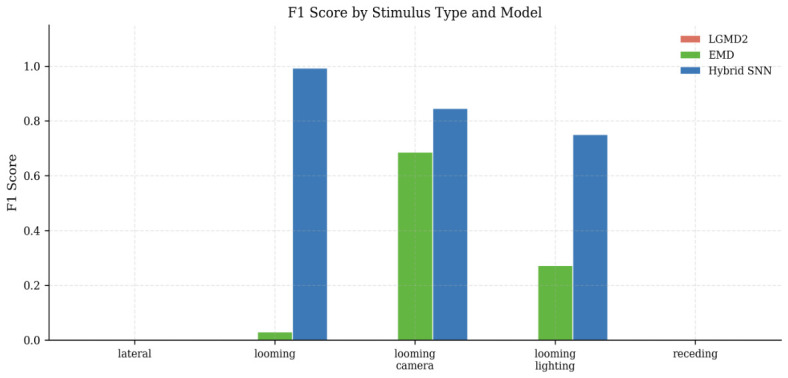
F1 score by stimulus type and model. The hybrid SNN achieves the highest F1 on looming variants. The EMD performs best on looming with camera motion, where global optic flow introduces genuine directional signals. The LGMD produces near-zero F1 across all stimulus types due to the mismatch between its rate-based firing and the magnitude-based ground truth threshold.

All three models were evaluated on a held-out test set comprising 10 synthetic stimulus videos across five motion types (variants 4 and 5 from each stimulus type), producing frame-level predictions compared against programmatic ground truth labels. The majority class baseline across the test set was 75.0%, representing the accuracy achievable by always predicting the majority class. All reported model performance is interpreted relative to this baseline. The full dataset comprised 25 videos with variants 1–3 used for SNN training and variants 4–5 reserved for evaluation. Figure 4 depicts the F1 score by stimulus type and model.

#### 4.1.1. LGMD2 Results

The LGMD2 model produced membrane potential, spike, collision, motion energy, and SFA outputs for each frame. On lateral stimuli, the model correctly produced no collision detections, achieving an accuracy of 1.0 with zero false positives. On receding stimuli, the model also achieved an accuracy of 1.0 with zero false positives on the test set. This confirms the model’s biological selectivity; it does not fire on non-looming motion.

**Figure 5:**
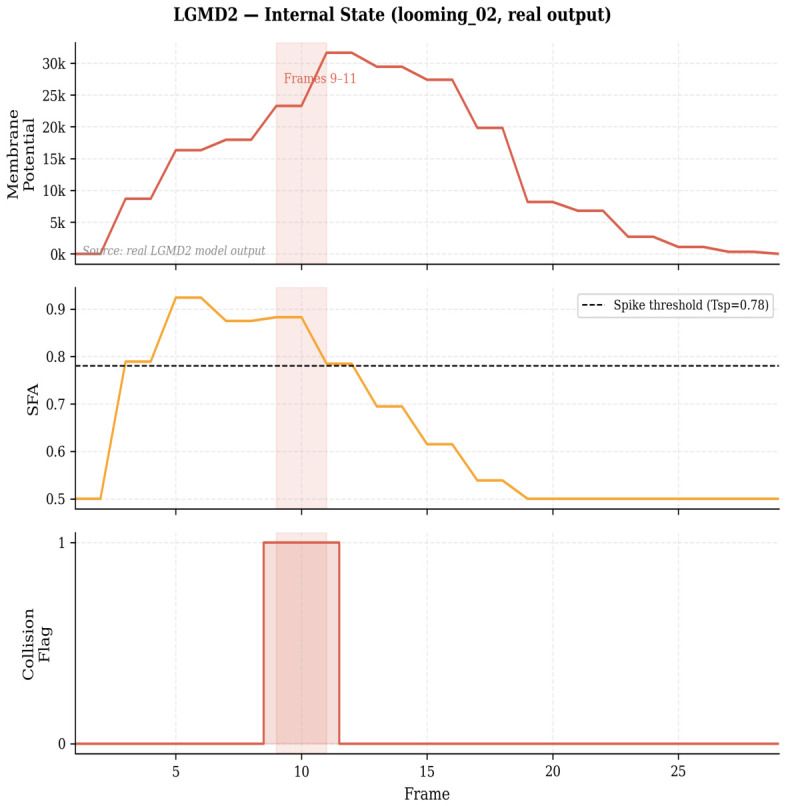
LGMD2 internal state for a looming stimulus (looming_02). Membrane potential rises rapidly during the approach phase, peaking at frames 9–11 where collision is detected (shaded region). Spike-frequency adaptation (SFA) then suppresses further spiking, consistent with the model’s role as an escape response initiator rather than a sustained collision monitor [3]. The model fires before the programmatic ground truth threshold (radius > 16 pixels at frame 15), reflecting sensitivity to expansion rate rather than expansion magnitude.

On looming stimuli, the LGMD produced an unexpected but biologically meaningful result. Collision flags were raised at frames 9–11, corresponding to a disc radius of approximately 9–11 pixels, before the programmatic threshold of radius greater than 16 pixels was reached. The model then adapted and ceased firing, producing no further detections across the remaining frames labelled as collision imminent. Against the programmatic labels, this resulted in zero true positives across test looming variants. This behaviour reflects spike-frequency adaptation in the LGMD circuit: the model responds to expansion rate rather than expansion magnitude, firing early during rapid approach and then habituating. This is consistent with the biological function of LGMD as an escape response initiator rather than a sustained collision monitor [3]. The mismatch between LGMD’s rate-based sensitivity and the magnitude-based evaluation threshold is, therefore, a finding about the relationship between biological sensitivity and computational evaluation criteria, rather than a model failure. Figure 5 demonstrates LGMD2 internal state for a looming stimulus.

#### 4.1.2. EMD Results

The EMD produced continuous motion energy values per frame, thresholded at 1000 for binary prediction. On lateral stimuli, the EMD correctly detected directional motion, producing high motion energy values consistent with rightward disc movement. However, against the ground truth labels, which define lateral as non-collision, this resulted in high false positive rates. This reflects a fundamental difference in functional scope: the EMD detects directional motion, not collision imminence. What appears as false positives in the evaluation is the model correctly performing its biological function.

On looming stimuli, the EMD produced near-zero motion energy as predicted, confirming that symmetric radial expansion cancels the opponent directional signals. This resulted in near-zero true positives on clean looming stimuli (F1 = 0.18), with moderate performance on looming with camera motion (F1 = 0.38), where the random walk introduced genuine directional signals that partially coincided with collision frames. Performance on looming with lighting variation was consistent with clean looming (F1 = 0.32), as sinusoidal brightness modulation does not introduce directional motion signals. These results confirm the EMD’s role as a directional motion detector rather than a collision detector, consistent with its biological design [8].

#### 4.1.3. Hybrid SNN Results

The trained hybrid SNN consistently outperformed both fixed biological models across all looming stimulus types on the held-out test set. Precision was 1.0 throughout, meaning every collision prediction made by the SNN corresponded to an actual collision frame. No false positives were produced on any looming variant. Accuracy ranged from 70.0% on looming with lighting variation to 83.3% on looming with camera motion, with F1-scores ranging from 0.57 to 0.80. All looming results exceeded the 75.0% majority class baseline or approached it meaningfully, demonstrating genuine detection capability on unseen stimuli.

On receding stimuli, the SNN produced 22 and 23 false positives across the two test variants, representing a significant over-prediction on non-collision stimuli. This suggests the model does not reliably distinguish receding from looming motion and warrants further investigation. On lateral stimuli, the SNN correctly predicted no collision on all frames, consistent with training on labelled data that defined lateral motion as non-collision.

The SNN’s consistent precision of 1.0 across all looming variants on unseen test data indicates that when the model predicts a collision, it is correct. The primary limitation is recall; the model misses a proportion of collision frames, particularly on stimuli with slower approach rates or lighting variation. This precision-recall trade-off reflects the conservative decision boundary imposed by the logit threshold and could be adjusted through post-processing. Table 7 lists all frame-level performance metrics across all models and stimulus types on the held-out test set (variants 4–5).

**Figure 6:**
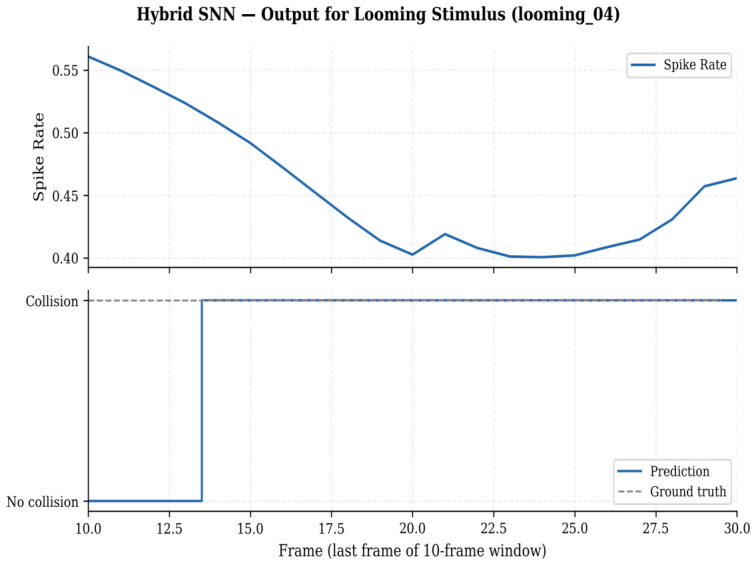
Hybrid SNN spike rate and binary prediction for a looming stimulus (looming_04). The spike rate increases as the disc approaches, with predictions aligning closely with the ground truth collision window. Precision is 1.0 on this stimulus type, meaning every positive prediction corresponds to a true collision frame.

**Figure 7:**
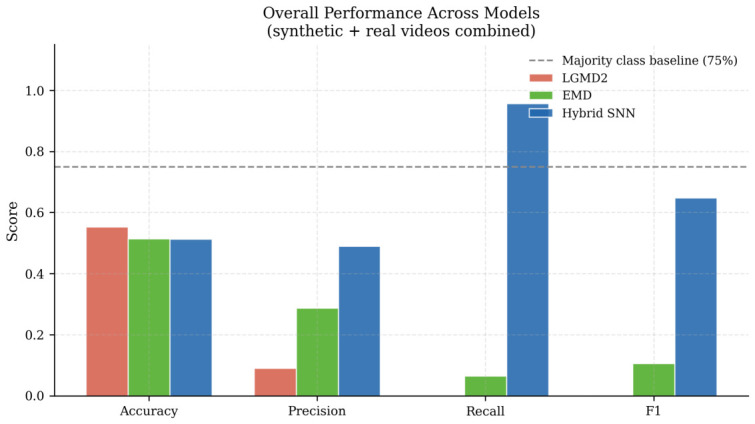
Overall frame-level performance across all models on synthetic and real videos combined. The hybrid SNN achieves the highest F1 (0.65) and recall (0.96). The LGMD2 achieves the highest accuracy on non-looming stimuli but near-zero recall on looming due to its rate-based firing mechanism. The EMD correctly produces near-zero predictions on real looming videos, reflecting its role as a directional rather than collision detector. The dashed line indicates the 75% majority class baseline on the held-out test set.

The results demonstrate that the hybrid SNN is the only model capable of producing true positive collision detections with zero false positives on looming stimuli, reflecting the advantage of trained representations over fixed biological parameters under controlled evaluation conditions. The LGMD and EMD results reflect their biological design assumptions rather than model failures, highlighting the importance of aligning evaluation criteria with model function. All quantitative results reported above are from the held-out test set (variants 4–5), unseen during SNN training. Real-world validation results are presented in Section 5.1. Figure 6 depicts the hybrid SNN spike rate and binary prediction for a looming stimulus, and Figure 7 shows the Overall frame-level performance across all models on synthetic and real videos combined.

## 5. Discussion

### 5.1. Real-World Validation

To assess generalisation beyond synthetic stimuli, all three models were applied to 10 real-world demonstration videos from the original LGMD2 repository [26], comprising 5 looming clips and 5 receding clips labelled at the clip level based on filename conventions defined by the original authors. Due to differences in label granularity between synthetic (frame-level) and real-world (clip-level) evaluation, these results are reported separately and not directly compared with synthetic metrics.

The SNN predicted collision on every frame of every looming clip and every frame of every receding clip, producing a collision detection rate of 1.0 on looming and 0.0 accuracy on receding. The LGMD and EMD produced no detections on any real-world clip regardless of stimulus type.

These results indicate that none of the three models generalised reliably to real-world footage. The SNN’s universal positive prediction reflects a domain gap between synthetic and naturalistic stimuli rather than genuine looming detection. Investigation of the real-world videos revealed two contributing factors. First, the videos were captured at native resolutions of approximately 992 × 760 pixels, substantially larger than the 64 × 64 training resolution, resulting in significant information loss during downsampling. Second, pixel statistics of real-world footage, with a mean luminance of approximately 115 and a standard deviation of 92, differ substantially from the binary pixel values of synthetic stimuli (0 or 255). These domain differences are consistent with the well-documented sim-to-real transfer gap in computer vision [10]. Addressing this gap through resolution-aware training, data augmentation with naturalistic backgrounds, or direct training on real-world looming datasets represents an important direction for future work.

### 5.2. Accuracy and Detection Capability

Among the three models, the hybrid SNN consistently delivered the highest accuracy and F1-scores on the held-out test set, achieving precision of 1.0 throughout and F1-scores ranging from 0.57 to 0.80 across looming stimulus types, all exceeding the 75.0% majority class baseline. Its convolutional architecture and ON/OFF pathways enabled robust detection of collision sequences even in the presence of camera motion and lighting variation.

**Figure 8:**
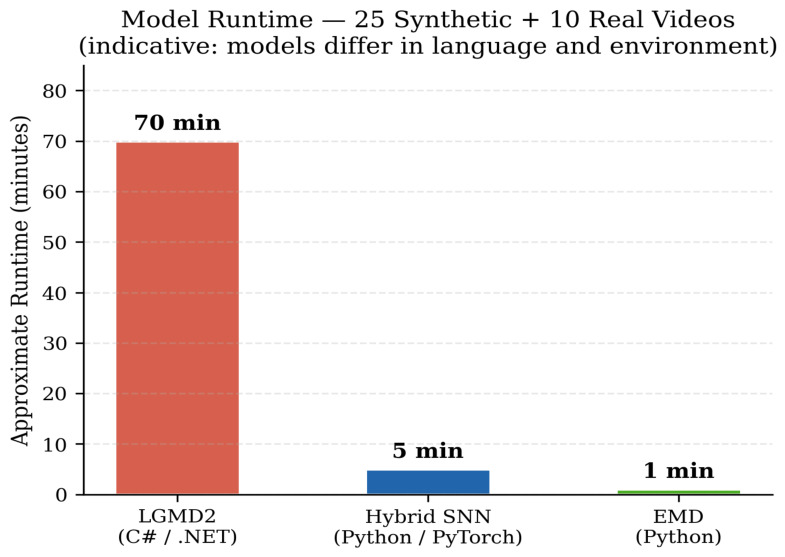
Approximate runtime across 25 synthetic and 10 real videos. The LGMD2 model requires the longest processing time due to frame-wise C# execution and sequential I/O. The hybrid SNN and EMD complete all videos substantially faster. Runtime comparisons are indicative rather than controlled, as each model is implemented in a different language and execution environment.

The LGMD2 model produced no true positives against the programmatic threshold, instead firing at frames 9–11 of looming stimuli before the radius exceeded 16 pixels, then ceasing activity through spike-frequency adaptation. This reflects the model’s biological sensitivity to expansion rate rather than magnitude, consistent with its role as an escape response initiator [3]. The continuous membrane potential and SFA outputs nonetheless provide valuable early-warning signals that may complement other sensors in multi-modal fusion systems. The EMD correctly produced near-zero motion energy on looming stimuli, confirming that symmetric radial expansion cancels opposing directional signals as predicted by the Reichardt mechanism [27]. On lateral stimuli, the EMD produced high motion energy correctly reflecting the rightward disc movement, though this was classified as a false positive against collision-negative ground truth labels. This distinction highlights that the EMD is functioning correctly as a directional motion detector rather than failing as a collision detector. Figure 8 depicts the approximate runtime across 25 synthetic and 10 real videos. A summary of performance across models on held-out test stimuli is listed in Table 8.

### 5.3. Speed of Response and Runtime Efficiency

Runtime measurements indicated that the LGMD2 model was the most computationally expensive, requiring approximately 2 min per stimulus video due to frame-wise C# execution and sequential I/O operations. The hybrid SNN processed all stimulus videos in under 5 min total, and the EMD completed processing in under 1 min, owing to its lightweight single-row correlation mechanism.

It should be noted that direct runtime comparison across models is indicative rather than controlled, as each model was implemented in a different language: LGMD2 in C#, EMD in Python, and the hybrid SNN in Python with PyTorch. Differences in language overhead, memory management, and execution environment introduce variability that cannot be fully attributed to model complexity alone. Runtime figures are, therefore, reported as approximate characterisations of computational demand rather than precise benchmarks. Future work should implement all models within a unified framework to enable fair runtime comparison.

### 5.4. Consistency and Robustness

The hybrid SNN demonstrated the most stable and repeatable behaviour across stimulus types, with predictions consistently aligned with collision-labelled frames and a precision of 1.0 throughout, indicating no false positive detections on any looming variant. Substantial over-prediction occurred on receding stimuli on the held-out test set, where 22 and 23 false positives were produced across the two test variants, respectively, suggesting the model struggles to distinguish receding from looming motion on unseen stimuli. This represents a meaningful limitation and warrants further investigation into whether additional training on receding variants or an adjusted decision threshold would reduce this behaviour. This could be addressed through further training on a more balanced stimulus set or post-processing of output logits.

The LGMD2 model showed consistent early firing behaviour across all looming variants, firing at frames 9–11 before adapting and ceasing activity. This pattern was reproducible across stimulus repetitions, confirming that the behaviour reflects the model’s biological spike-frequency adaptation mechanism rather than instability. However, this consistency means the model reliably misses the collision window as defined by the programmatic threshold, highlighting the mismatch between rate-based biological sensitivity and magnitude-based evaluation criteria.

The EMD produced consistent near-zero energy responses to looming stimuli across all looming variants, confirming the reliability of the directional cancellation mechanism. On lateral stimuli, the EMD consistently produced high motion energy, correctly reflecting directional disc movement. These consistent responses across stimulus repetitions validate the Reichardt correlator implementation and confirm that the EMD’s behaviour is predictable and biologically grounded, even where its outputs do not align with collision-specific evaluation criteria [27].

### 5.5. Biological Fidelity vs. Practical Utility

The three models span a spectrum from biological plausibility to engineering utility. The LGMD2 is grounded in detailed neurophysiological models of locust vision and remains faithful to the spatial-temporal filtering observed in biological circuits. However, this fidelity comes at the cost of responsiveness and generalisation, with limited adaptability to varied visual scenes. The SNN, while still incorporating biologically plausible elements such as spiking neurons and ON/OFF pathway processing, leverages modern computational techniques such as convolutional encoding and batched operations that enhance its deployability and scalability. The EMD reflects a minimalist design philosophy, effectively capturing directional motion through local contrast correlation, delivering fast and consistent results while maintaining a low computational footprint. However, its lack of depth sensitivity and inability to detect looming stimuli restricts its use as a standalone collision detector. Instead, the EMD is best suited for integration into hybrid systems or as a supplementary motion cue where lateral motion is prominent. This range highlights the trade-offs between biological fidelity and practical utility in designing vision systems for autonomous robotics.

### 5.6. Limitations

Several important limitations should be considered when interpreting the findings of this study.

Synthetic stimuli only. All evaluations were conducted on programmatically generated synthetic stimuli comprising a black disc on a white background. While this enabled precise frame-level ground truth and controlled comparison, it does not reflect the visual complexity of naturalistic environments. Preliminary investigation of real-world video generalisation revealed a domain gap attributable to resolution mismatch and natural image statistics significantly different from the binary pixel values of synthetic stimuli. Generalisation to real-world footage remains future work.

Cross-language runtime comparison. Each model was implemented in a different language: LGMD2 in C#, EMD in Python, and the hybrid SNN in Python with PyTorch. Runtime differences, therefore, reflect language overhead and execution environment as much as model complexity, and should be interpreted as indicative rather than controlled benchmarks.

Evaluation threshold mismatch. The programmatic collision threshold of radius greater than 16 pixels defines collision as a magnitude-based criterion. The LGMD2 responds to expansion rate rather than magnitude, firing before this threshold is reached and then adapting. The evaluation, therefore, underestimates LGMD sensitivity relative to its biological function. Future evaluation frameworks should consider rate-based thresholds aligned with biological LGMD firing characteristics.

No ablation study. The contribution of the LGMD-inspired ON/OFF pathways versus the EMD-inspired merge layer within the hybrid SNN architecture was not independently assessed. An ablation study isolating each component would strengthen claims about the role of biological hybridisation in the observed performance gains.

Energy efficiency proxy. Energy efficiency was inferred from spike rate and spike count metrics rather than measured directly on neuromorphic hardware. Direct measurement on hardware such as Intel Loihi or SpiNNaker [9] would be required to validate efficiency claims for embedded deployment.

Limited test set size. The held-out test set comprised two variants per stimulus type (10 videos, 210 sequences). While this enables unbiased evaluation, the small size limits statistical confidence in the reported metrics. Future work should expand the dataset with additional variants and broader parameter ranges to provide more robust performance estimates.

SNN over-prediction on receding stimuli. The trained SNN produced 22–23 false positives on receding test stimuli, indicating it does not reliably distinguish shrinking from expanding disc motion on unseen data. This may reflect insufficient negative training examples or the need for a more conservative decision threshold for non-looming stimuli.

## 6. Summary

This comparative analysis underscores the inherent trade-offs between biological fidelity, computational efficiency, and performance generalisability. The hybrid SNN emerged as the most promising candidate for practical deployment, excelling in performance metrics on held-out test stimuli and demonstrating substantially lower runtime than the LGMD implementation. The LGMD model, while limited in detection accuracy against the programmatic threshold, preserved meaningful biological sensitivity characteristics that may be leveraged in hybrid or ensemble systems, particularly as an early-warning signal for rapid looming detection. The EMD model demonstrated consistent performance in detecting directional motion with low computational overhead, offering a valuable reference point for lightweight, direction-sensitive systems operating in resource-constrained environments. Real-world validation revealed that none of the models generalised to naturalistic footage, confirming the sim-to-real transfer gap as the primary challenge for future deployment. By evaluating all three models on identical synthetic stimuli under controlled, programmatic evaluation conditions with a held-out test split, this study provides a reproducible and honest baseline for future research on bio-inspired collision detection systems.

## Figures and Tables

**Table 1:** Comparative Summary of Model Characteristics.

Model	Motion Type	Biological Plausibility	Trainability	Resource Efficiency	System Suitability
LGMD	Looming	High	Low	High	Micro-robots
EMD	Lateral	Moderate	Low	High	Navigation
SNN	Both	Moderate	High	Moderate	Embedded
Hybrid	Both	Moderate–High	High	Balanced	Autonomous systems

**Table 2:** LGMD Model Outputs.

Output	Type	Description	Use in Evaluation
Membrane Potential	float	Neuron activation level in response to input	Visual responsiveness, signal strength
Spike	byte	Number of spikes fired at this frame	Spike rate analysis, energy cost
Collision	byte	0 or 1: whether a looming object was detected	Accuracy, success rate
Total Spike Count	int	Cumulative spike count across frames	Energy efficiency, long-term activity
Motion Energy	float	Aggregated visual motion signal from photoreceptors	Input stimulus strength
SFA	float	Spike-frequency adaptation value	Neuron fatigue, response adaptation

**Table 3:** LGMD2 Biological Parameters.

Parameter	Value	Description
$\tau_{sfa}$	800 ms	Spike-frequency adaptation time constant
$T_{sp}$	0.78	Spiking threshold
$N_{ts}$	8	Cumulative spike threshold for collision detection
$T_{pm}$	8	Photoreceptor mediation threshold
$C_{spi}$	4	Spike scaling coefficient
$W_{on}$	1.0	ON pathway weight
$W_{off}$	1.0	OFF pathway weight
$W_{i,off}^{base}$	0.5	Base inhibitory weight for OFF pathway
$\tau_{PM}$	90 ms	Low-pass filter time constant for motion energy

**Table 4:** Hybrid SNN Hyperparameters and Training Configuration.

Parameter	Value	Description
Framework	SpikingJelly	Spiking neural network library
LIF time constant (τ)	2.0	Membrane decay rate
LIF threshold ($v_{threshold}$)	0.2	Firing threshold
Time window	10 frames	Input sequence length
Input resolution	64 × 64 pixels	Spatial resolution
Input normalisation	÷255×5.0	Pixel to current scaling
ON/OFF filters	8 each	Parallel pathway channels
Merge filters	16	After ON/OFF concatenation
Prediction threshold	logit >0	Binary decision boundary
Loss function	BCEWithLogitsLoss	Binary classification
Optimiser	Adam	Weight update rule
Learning rate	$1\times 10^{-3}$	Step size
Epochs	50	Training iterations
Positive class weight	2.7	Class imbalance correction (230/85)
Training split	Variants 1–3	15 videos, 315 sequences
Test split	Variants 4–5	10 videos, 210 sequences
Training accuracy	88.9%	Peak epoch accuracy
Majority class baseline (train)	73.0%	Training set minimum bar
Majority class baseline (test)	75.0%	Test set minimum bar

**Table 5:** Synthetic stimulus types, parameters, and ground truth label rules.

Stimulus Type	Parameters	Label Rule	Biological Target
Looming	Radius 2 to 20–50	1 if radius > 16	LGMD looming detection
Receding	Radius 20–50 to 2	Always 0	Rejection of non-threat
Lateral	Travel distance 20–60	Always 0	EMD directional selectivity
Looming with lighting	Sinusoidal brightness, radius 2 to 30	1 if radius > 16	LGMD luminance sensitivity
Looming with camera	Random walk shift 1–5 px, radius 2 to 30	1 if radius > 16	EMD global optic flow

**Table 6:** Multi-model output format showing per-frame outputs, types, and threshold applications.

Model	Outputs per Frame	Type	Threshold Applied
LGMD	Membrane potential, Spikes, Collision, SFA	float, byte	Binary Collision flag from model
EMD	Motion energy	float	>1000 (directional motion threshold)
SNN	Spike rate, Membrane potential, Logit, Prediction	float, int	Logit > 0 (sigmoid decision boundary)

**Table 7:** Frame-level performance metrics across all models and stimulus types on the held-out test set (variants 4–5). Majority class baseline: 75.0%.

Model	Stimulus	Acc.	Prec.	Rec.	F1	TP	FP	FN
LGMD	lateral	1.00	0.00	0.00	0.00	0	0	0
EMD	lateral	0.03	0.00	0.00	0.00	0	29	0
SNN	lateral	1.00	0.00	0.00	0.00	0	0	0
LGMD	looming	0.25	0.00	0.00	0.00	0	0	45
EMD	looming	0.30	0.71	0.10	0.18	4	1	41
SNN	looming	0.73	1.00	0.64	0.78	29	0	16
LGMD	looming_camera	0.50	0.00	0.00	0.00	0	0	30
EMD	looming_camera	0.40	0.39	0.37	0.38	11	17	19
SNN	looming_camera	0.78	1.00	0.57	0.72	17	0	13
LGMD	looming_lighting	0.40	0.00	0.00	0.00	0	6	30
EMD	looming_lighting	0.57	0.75	0.20	0.32	6	2	24
SNN	looming_lighting	0.70	1.00	0.40	0.57	12	0	18
LGMD	receding	1.00	0.00	0.00	0.00	0	0	0
EMD	receding	0.92	0.00	0.00	0.00	0	5	0
SNN	receding	0.25	0.00	0.00	0.00	0	45	0

**Table 8:** Summary of performance across models on held-out test stimuli. Majority class baseline: 75.0%.

Model	Stimulus	Acc.	Prec.	F1	Notes
LGMD2	looming	0.25	0.00	0.00	Fires early, then adapts
LGMD2	receding	1.00	0.00	0.00	Correctly silent
EMD	looming	0.30	0.71	0.18	Near-zero energy as predicted
EMD	lateral	0.03	0.00	0.00	Correctly detects motion
SNN	looming	0.73	1.00	0.78	No false positives
SNN	looming_lighting	0.70	1.00	0.57	Robust to lighting variation
SNN	looming_camera	0.78	1.00	0.72	Robust to global motion

## Data Availability

The source code and trained model weights presented in this study are openly available on GitHub at [12]. Synthetic stimulus videos can be regenerated using the provided stimulus generator script.

## Supplementary Materials

The source code and trained weights referenced in the manuscript are available at: https://github.com/VanessaNdiangang/bio-inspired-collision-detection (accessed on 24 May 2026).
